# Health-related quality of life for medical rescuers one month after Ludian earthquake

**DOI:** 10.1186/s12955-015-0286-5

**Published:** 2015-06-25

**Authors:** Bihan Tang, Yang Ge, Zhipeng Liu, Xu Liu, Peng Kang, Yuan Liu, Lulu Zhang

**Affiliations:** Institute of Military Health Management, Second Military Medical University, 800 Xiangyin Rd, Shanghai, 200433 China

**Keywords:** Quality of life, Earthquake, Medical rescuers, Risk factor

## Abstract

**Background:**

An earthquake struck Ludian in Yunnan province of China on August 3, 2014, resulting in 3143 injuries, 617 deaths, and 112 missing persons. Our study aimed at estimating the quality of life and associated determinants among medical rescuers after Ludian earthquake.

**Methods:**

A cross-sectional survey was performed among personnel from three hospitals that assumed rescue tasks in Ludian earthquake. Descriptive statistics, t-tests, ANOVA and stepwise linear regression analysis were used for data analysis.

**Results:**

The mean scores on the physical component summary (PCS) and mental component summary (MCS) were 49.86 (SD = 6.01) and 35.85(SD = 6.90), respectively. Lower PCS in the aftermath of an earthquake was associated with non-military medical rescuers, elderly age, and being trapped/in danger while lower MSC in the aftermath of an earthquake was associated with non-military medical rescuers, young age, being female, being trapped/in danger and low education degree.

**Conclusions:**

In conclusion, our study demonstrates that medical rescuers are at risk for a lower HRQoL after exposure to Ludian earthquake. The results of this study help expand our knowledge of health-related quality of life among medical rescuers after the Ludian earthquake.

## Introduction

On Sunday, August 3, 2014, an earthquake of 6.5 on the magnitude scale and 8.0 on the intensity scale struck Ludian in Yunnan province of China at 16:30 h local time. Often referred to as Ludian earthquake, this disaster resulted in 3143 injuries, 617 deaths, and 112 missing persons. Thousands of rescue workers from the military and medical system were sent into the disaster areas in a few hours. Those who were injured during the earthquake were sent to the nearest local hospitals immediately. However, since some local clinics and health centres were damaged during the earthquake, part of the medical rescue was also operated in temporary facilities.

Medical rescue workers had to face increasing physical and psychological demands under life-threatening conditions (such as landslides and aftershocks) [1]. They were exposed to terrifying scenes of dead or severely wounded people; screams of people wanting help; and the mourning, impatience, and rowdiness of people who had relatives or property under the wreckage [2]. Moreover, they also experienced extended work hours, lack of sleep, and harsh living conditions in makeshift quarters. In addition to acting as helpers, medical rescuers may also become hidden victims and should receive particular attention.

Over the past several decades, researchers have become aware that rescuers who are exposed to massive structural disruption and casualties during natural disasters suffer from physical complaints and mental disorders because of the physical and psychological stresses endured during these events [3]. Several studies found that posttraumatic stress disorder (PTSD) was popular among rescue workers involved in major disasters [2, 4–6]. Other mental health disorders have also been described, including depression and anxiety [7–10]. However, most previous studies only focused on the mental health status of rescue workers but ignored their physical health status. Only one study discussed health-related quality of life (HRQoL) among the rescuers after an air disaster in Amsterdam, and discussed their physical and mental health status at the same time [11]. The concept of HRQoL, focusing on an individual’s physical, mental, and social well-being status, [12] has emerged as one of the most important measurements in clinical trials and population health surveys [13, 14]. However, to our knowledge, no cross-sectional study has examined the HRQoL of rescue workers after earthquakes. In this study, we aimed to discuss the HRQoL and related risk factors of medical workers one month after participating in rescue work in Ludian earthquake, and thus provide evidence-based suggestions for specific health interventions in vulnerable populations.

## Methods

### Study design

A cross-sectional survey was performed in September 2014, by which time one month had passed since the event and the medical rescue tasks had been finished. Personnel from three hospitals that assumed rescue tasks in Ludian earthquake participated in the present study, including People’s Hospital located in Ludian County, about 31 km from the epicentre of the Ludian earthquake. People’s Hospital is the biggest hospital in Ludian county and received a majority of the victims injured in this earthquake [15]. The other two were military hospitals located in Kaiyuan and Kunming of Yunnan Province, respectively. These two military hospitals dispatched medical rescue teams to Ludian county no more than two hours after the earthquake occurred, which functioned as temporary hospitals and received the injured in the stricken areas. The ethical committee of the Second Military Medical University approved the study protocol.

### Data collection

Five researchers were dispatched to Yunnan province to conduct this investigation in these three hospitals. Anonymity and confidentiality were guaranteed, and informed consent from the participants was required. The data were collected by self-administered anonymous paper questionnaires, which were reviewed by experts from the research fields of health services and disaster medicine. Inter-rater reliability was assessed until 100 % concordance in use of the instrument was achieved among the interviewers [16]. A total of 468 medical rescue personnel involved in the rescue task of this earthquake were contacted in this study. Ninety-six failed to participate in this investigation because they were on vacation at that time. Thus, 372 people completed our questionnaires, and 23 questionnaires were excluded because of missing items. The total number of completed questionnaires was 349.

### Measures

Questionnaires covered the following categories: age, marital status, gender, ethnicity, education level, occupation, professional title, military medical rescuers or not, trapped/in danger or not, having rescue training or not, and involved in previous rescue or not. We classified the rescuers’ professional titles into four categories: no, junior (resident doctors), intermediate (attending doctors) and senior (associate chief doctors or chief doctors).

The HRQoL of the rescue workers was measured using the 12-item short form health survey (SF-12), which is a practical, reliable, and valid measurement. The SF-12 is a subset of the 36-item short form health survey (SF-36) scale and is one of the most commonly used HRQoL questionnaires. Translated into multiple languages, the SF-12 includes two from each of the physical functioning, role-physical, role-emotional, and mental health scales and one item from each of the bodily pain, general health, vitality, and social functioning scales of the SF-36 [17]. In addition, the SF-12 assesses overall physical and mental function using summary scales, physical component summary (PCS), and mental component summary (MCS) [18]. Higher summary PCS and MCS scores are indicative of better health. Scores above or below 50 are considered to be above and below the average in the general population [19]. The Chinese version of the SF-12 used in this study has been validated in the Chinese population [17].

### Data analysis

All analyses were performed *via* a statistical software package (Statistical Package for the Social Sciences/SPSS Version 11.0). We first calculated the descriptive statistics (frequencies, percentages, means, and standard deviations [SD]); then, t-tests (for two-group comparison) and analysis of variance (ANOVA, for multi-group comparison) were used to evaluate differences in continuous variables if the data were in accordance with the normality distribution and homogeneity of variance; otherwise, a non-parametric method was used. Stepwise linear regression analysis was performed to identify predictors of PCS and MCS, and beta coefficients (B), standardized error of coefficient (S.E.B), and standardized regression coefficient (Beta) were reported. The criterion for statistical significance was P = 0.05.

## Results

### Social-demographic characteristics and earthquake-related experiences

The total number of completed questionnaires was 349. These results are presented in Table 1. The mean scores of the PCS and MCS were 49.86 (SD = 6.01) and 35.85 (SD = 6.90), respectively. Of the 349 participants, 108 (30.95 %) were military medical rescuers, the mean of age was 34.8 (19–60), 198 (56.73 %) were female, about two thirds of the participants (234, 67.05 %) were married, 270 (77.36 %) were of Han descent (the major ethnic population in China), 159 (45.56 %) had an undergraduate degree or above, 125 (35.82 %) and 156 (44.70 %) were doctors and nurses, and most participants had junior (173, 49.57 %) or intermediate (121, 34.67 %) professional titles. With regard to earthquake related experiences, 78 (22.35 %) were trapped or in danger in the earthquake, most participants had received rescue training (305, 87.39 %) and had been involved in previous rescues (244, 69.91 %).

**Table 1:** Demographics and earthquake-related experiences among rescuers of the Ludian earthquake, China

Variable	N	Percentage (%)	PCS		MSC	
			Mean	P	Mean	P
Total	349	-	49.86 (6.01)	-	35.85 (6.90)	-
Military medical rescuer				0.002		<0.001
No	241	69.05	49.2 (6.25)		34.63 (6.66)	
Yes	108	30.95	51.32 (5.16)		38.58 (6.67)	
Age				0.039		<0.001
<30	102	29.23	51.08 (5.09)		34.48 (6.23)	
30–40	158	45.27	49.72 (6.60)		35.52 (7.48)	
>40	89	25.50	48.70 (5.68)		38.02 (6.08)	
Marriage				0.089		0.332
No	115	32.95	50.75 (5.56)		35.38 (6.31)	
Yes	234	67.05	49.42 (6.18)		36.08 (7.18)	
Gender				0.347		<0.001
Male	151	43.27	49.38 (6.61)		37.72 (7.25)	
Female	198	56.73	50.22 (5.49)		34.43 (6.28)	
Ethnicity				0.028		0.176
Minority	79	22.64	48.87 (5.56)		35.04 (6.91)	
Han	270	77.36	50.15 (6.11)		36.09 (6.89)	
Education level				0.056		<0.001
Graduate	35	10.03	50.47 (7.18)		39.03 (7.44)	
Undergraduate	124	35.53	49.59 (6.21)		36.65 (6.77)	
College	97	27.79	48.96 (6.36)		36.25 (7.31)	
Other	93	26.65	50.93 (4.62)		33.18 (5.54)	
Occupation				0.278		0.411
Doctor	125	35.82	49.82 (5.77)		36.05 (6.88)	
Nurse	156	44.70	50.55 (5.22)		35.47 (7.18)	
Other	68	19.48	48.33 (7.70)		36.37 (6.29)	
Professional titles				0.013		0.460
No	40	11.46	50.43 (6.35)		34.19 (6.42)	
Junior	173	49.57	50.71 (5.44)		36.04 (6.98)	
Intermediate	121	34.67	48.53 (6.29)		36.21 (7.13)	
Senior	15	4.3	49.13 (7.52)		35.3 (4.92)	
Trapped/in danger				0.339		0.095
No	271	77.65	50.18 (5.51)		36.21 (6.79)	
Yes	78	22.35	48.72 (7.41)		34.63 (7.17)	
Rescue training				0.685		0.188
No	44	12.61	49.67 (5.91)		36.94 (6.89)	
Yes	305	87.39	49.88 (6.03)		35.7 (6.9)	
Involved in previous rescue				0.798		<0.001
No	105	30.09	50.04 (5.38)		37.58 (7.25)	
Yes	244	69.91	49.78 (6.27)		35.11 (6.62)	

### Factors associated with PCS

Bivariate analyses (Table 1) were conducted to assess quality of life during the aftermath of an earthquake, as assessed by the PCS. In Table 1, PCS were significantly associated with Military medical rescuers (P = 0.002), age (P = 0.039), ethnicity (P = 0.028), and professional title (P = 0.013). Multiple stepwise regression analyses (Table 2) revealed that lower PCS in the aftermath of an earthquake was associated with non-military medical rescuers (P = 0.001), old age (P < 0.001), and being trapped/in danger (P = 0.017).

**Table 2:** Multiple stepwise regression analysis of factors independently associated with PCS and MCS

Label	b^a^	S.E.B^b^	B^c^	P^d^
Dependent variable: PCS				
Age	−0.129	0.035	−0.192	0.000
Military medical rescuers	2.273	0.677	0.175	0.001
Trapped or in danger	−1.800	0.751	−0.125	0.017
Dependent variable: MCS				
Military medical rescuers	3.208	0.811	0.215	0.000
Age	0.121	0.039	0.156	0.000
Gender	−1.573	0.746	−0.113	0.002
Trapped/in danger	−2.027	0.833	−0.123	0.036
Education level	−0.884	0.390	−0.124	0.015

^a^Unstandardized regression coefficient

^b^Standardized error of coefficient

^c^tandardized regression coefficient

^d^The inclusion criteria of the stepwise regression was P = 0.05; exclusion criteria was P = 0.10

### Factors associated with MCS

Bivariate analyses (Table 1) were conducted to assess the quality of life during the aftermath of an earthquake, as assessed by the MCS. In Table 1, MCS were significantly associated with military medical rescuers (P < 0.001), age (P < 0.001), gender (P < 0.001), education degree (P < 0.001), and being involved in a previous rescue (P < 0.001). Multiple stepwise regression analyses (Table 2) revealed that lower MCS in the aftermath of an earthquake was associated with non-military medical rescuers (P < 0.001), young age (P < 0.001), being female (P = 0.002), being trapped/in danger (P = 0.023) and low education degree (P = 0.015).

## Discussion

Unlike many other natural disasters, earthquakes come without warning, and their impact is often widespread and their effects ongoing [20]. In the face of earthquakes and corresponding harsh environments, rescuers may become fragile both physically and mentally [21]. Research into the HRQoL of medical rescuers could prove valuable data for future rescue work and enhance the overall effectiveness of rescue efforts after a major earthquake [22].

Using the SF-12 as an instrument to assess the impact of the Ludian earthquake rescue experience on the overall HRQoL of medical rescuers, we found that the rescuers had a similar PCS score compared to the normal population (49.86 *vs* 50); however, they had an obviously lower MCS score than the normal population (35.85 *vs* 50). This result confirmed that the HRQoL of rescuers, especially for mental health status, had been impaired by the Ludian earthquake. Interestingly, when we compare the health status of the rescuers with the survivors after Wenchuan earthquake, we found the rescuers had an obvious higher PCS score (49.86 *vs* 37.6) but a relatively low MCS score (35.85 *vs* 36.8) [23]. Medical rescuers’ duty is to provide professional medical care to the wounded as soon as possible. In executing their responsibilities, they may confront dead bodies, seriously injured victims, and negative reactions and even violence on the part of victims [24, 25]. All of these factors may contribute to the impairment of rescuers’ psychological health status.

With respect to risk factors of HRQoL among rescuers, we found that military medical rescuers had both higher PCS and MCS scores. As military service members, these medical personnel go to the nearest encampment and participate in a series of military skills training sessions every year. By training, they build good physical fitness and learn coping skills in the face of harsh conditions.

Being trapped/in danger is one factor that not only worsens rescuers’ physical health status (PCS: 48.72 *vs*. 50.18) but also puts them at greater risk for bad mental health status (MSC: 34.63 *vs*. 36.21). The government should pay more attention to rescuers placed in danger and ensure that when the rescuers get hurt, they receive medical and psychological services in a timely fashion. Age is another predictor that has significant impact on rescuers’ physical health status. This result is easy to understand since physical strength declined with age. However, the same trend didn’t occur for psychological aspects, which is inconsistent with previous studies [6, 9, 26].

As for risk factors for rescuers’ mental state, we found gender and education level were two important factors. Previous studies have indicated that women are more sensitive to threats, less likely to use effective coping strategies, and tend to interpret disasters more negatively than men do [27]. In addition, women are thought to be more sensitive to stress hormones, so their ability to manage stressful situations may be relatively poorer than men’s ability [28]. This finding suggests the need to pay more attention to women and create targeted interventions for female rescuers who respond to large disasters to reduce their psychological burden. Coping skills training (*e.g*. anger/hostility management and self-efficacy training) could be helpful for primary and secondary prevention in female rescuers [29]. Educational level indirectly influences economic resources, social status, social networks, health behaviour, and so on [30]. Medical rescuers with higher education levels might employ better coping methods because they have greater social resources, thus reducing the incidence of mental health problems.

Inconsistent with previous studies, we found that rescue training was not a significant predictor in our study [31]. This phenomenon may remind relevant policy makers to pay more attention to the content and quality of rescue training that medical personnel received before starting work. A series of comprehensive and systematic disaster relief training should be popularized among medical staff.

Certain limitations of this study should be recognized. First, this study is based on questionnaires; as such, it allows for the possibility of response bias. Another limitation is the study’s cross-sectional design. Longitudinal studies are needed preferably with an assessment prior to severe events. Besides, as an observational study, we took many factors into account, but might ignore some important confounders such as lifestyle, mental state, emotional component, co-existing stressors, mental state, coping strategies and so on. Despite these limitations, the present study is the first cross-sectional study focusing on health-related quality of life and its risk factors in medical rescue populations following an earthquake. In our study, significant associations between demographic, trauma-related, and rescue-related experiences variables and overall physical and mental health of medical rescuers were presented.

## Conclusions

In conclusion, our study demonstrates that medical rescuers are at risk for a lower HRQoL after exposure to Ludian earthquake. Establishing a sustainable qualified healthcare system for medical rescuers during and after earthquakes is of vital importance for future health crises. We also suggest improving pre-employment strategies to select the most resilient individuals for rescue work, implement continuous preventive measures for personnel, and promote educational campaigns about health problems and possible therapies. More systematic health services and training should be distributed to medical rescuers and at-risk groups to ensure they are adequately prepared for their participation in relief efforts. Further prospective studies should be undertaken to examine the cause-and-effect relationship between disasters and the outcome of health status.

## References

[1] Weiss DS, Marmar CR, Metzler TJ, Ronfeldt HM (1995). Predicting symptomatic distress in emergency services personnel. J Consult Clin Psychol.

[2] Ozen S, Sir A (2004). Frequency of PTSD in a group of search and rescue workers two months after 2003 Bingol (Turkey) earthquake. J Nerv Ment Dis.

[3] Berrios-Torres SI, Greenko JA, Phillips M, Miller JR, Treadwell T, Ikeda RM (2003). World Trade Center rescue worker injury and illness surveillance, New York, 2001. Am J Prev Med.

[4] Lesaca T (1996). Symptoms of stress disorder and depression among trauma counselors after an airline disaster. Psychiatr Serv.

[5] Marmar CR, Weiss DS, Metzler TJ, Delucchi K (1996). Characteristics of emergency services personnel related to peritraumatic dissociation during critical incident exposure. Am J Psychiatry.

[6] Chang CM, Lee LC, Connor KM, Davidson JR, Jeffries K, Lai TJ (2003). Posttraumatic distress and coping strategies among rescue workers after an earthquake. J Nerv Ment Dis.

[7] Elklit A (2007). Psychological consequences of a firework factory disaster in a local community. Soc Psychiatry Psychiatr Epidemiol.

[8] Marmar CR, Weiss DS, Metzler TJ, Ronfeldt HM, Foreman C (1996). Stress responses of emergency services personnel to the Loma Prieta earthquake Interstate 880 freeway collapse and control traumatic incidents. J Trauma Stress.

[9] Fullerton CS, Ursano RJ, Wang L (2004). Acute stress disorder, posttraumatic stress disorder, and depression in disaster or rescue workers. Am J Psychiatry.

[10] West C, Bernard B, Mueller C, Kitt M, Driscoll R, Tak S (2008). Mental health outcomes in police personnel after Hurricane Katrina. J Occup Environ Med.

[11] Slottje P, Twisk JW, Smidt N, Huizink AC, Witteveen AB, van Mechelen W (2007). Health-related quality of life of firefighters and police officers 8.5 years after the air disaster in Amsterdam. Qual Life Res.

[12] Jia Z, Tian W, He X, Liu W, Jin C, Ding H (2010). Mental health and quality of life survey among child survivors of the 2008 Sichuan earthquake. Qual Life Res.

[13] Erhart M, Ravens-Sieberer U (2006). Health-related quality of life instruments and individual diagnosis - a new area of application. Psycho-social medicine.

[14] Berzon R, Hays RD, Shumaker SA (1993). International use, application and performance of health-related quality of life instruments. Qual Life Res.

[15] NA: People Hospital. http://yyk.99.com.cn/ludian/88255/jianjie.html. 2014.

[16] Jia Z, Shi L, Duan G, Liu W, Pan X, Chen Y (2013). Traumatic experiences and mental health consequences among child survivors of the 2008 Sichuan earthquake: a community-based follow-up study. BMC Public Health.

[17] Lam CL, Tse EY, Gandek B (2005). Is the standard SF-12 health survey valid and equivalent for a Chinese population?. Qual Life Res.

[18] Liang Y, Cao R (2014). Is the health status of female victims poorer than males in the post-disaster reconstruction in China: a comparative study of data on male victims in the first survey and double tracking survey data. BMC Womens Health.

[19] Larson CO, Schlundt D, Patel K, Beard K, Hargreaves M (2008). Validity of the SF-12 for use in a low-income African American community-based research initiative (REACH 2010). Prev Chronic Dis.

[20] Guo YJ, Chen CH, Lu ML, Tan HK, Lee HW, Wang TN (2004). Posttraumatic stress disorder among professional and non-professional rescuers involved in an earthquake in Taiwan. Psychiatry Res.

[21] Witteveen AB, Bramsen I, Twisk JW, Huizink AC, Slottje P, Smid T (2007). Psychological distress of rescue workers eight and one-half years after professional involvement in the Amsterdam air disaster. J Nerv Ment Dis.

[22] Zhang L, Liu X, Li Y, Liu Y, Liu Z, Lin J (2012). Emergency medical rescue efforts after a major earthquake: lessons from the 2008 Wenchuan earthquake. Lancet.

[23] Kun P, Wang Z, Chen X, Le H, Gong X, Zhang L (2010). Public health status and influence factors after 2008 Wenchuan earthquake among survivors in Sichuan province, China: cross-sectional trial. Public Health.

[24] North CS, Tivis L, McMillen JC, Pfefferbaum B, Spitznagel EL, Cox J (2002). Psychiatric disorders in rescue workers after the Oklahoma City bombing. Am J Psychiatry.

[25] van der Velden PG, van Loon P, Benight CC, Eckhardt T (2012). Mental health problems among search and rescue workers deployed in the Haiti earthquake 2010: a pre-post comparison. Psychiatry Res.

[26] Chen HC, Chou FH, Chen MC, Su SF, Wang SY, Feng WW (2006). A survey of quality of life and depression for police officers in Kaohsiung, Taiwan. Qual Life Res.

[27] Zhang Z, Wang W, Shi Z, Wang L, Zhang J (2012). Mental health problems among the survivors in the hard-hit areas of the Yushu earthquake. PLoS One.

[28] Zhou X, Kang L, Sun X, Song H, Mao W, Huang X (2013). Risk factors of mental illness among adult survivors after the Wenchuan earthquake. Soc Psychiatry Psychiatr Epidemiol.

[29] Perrin MA, DiGrande L, Wheeler K, Thorpe L, Farfel M, Brackbill R (2007). Differences in PTSD prevalence and associated risk factors among World Trade Center disaster rescue and recovery workers. Am J Psychiatry.

[30] Lee J (2011). Pathways from education to depression. J Cross Cult Gerontol.

[31] Berger W, Coutinho ES, Figueira I, Marques-Portella C, Luz MP, Neylan TC (2012). Rescuers at risk: a systematic review and meta-regression analysis of the worldwide current prevalence and correlates of PTSD in rescue workers. Soc Psychiatry Psychiatr Epidemiol.

